# In-office diagnostic arthroscopy for knee and shoulder intra-articular injuries its potential impact on cost savings in the United States

**DOI:** 10.1186/1472-6963-14-203

**Published:** 2014-05-05

**Authors:** Jeffrey D Voigt, Michael Mosier, Bryan Huber

**Affiliations:** 1 99 Glenwood Road, 07450 Ridgewood, NJ, USA; 2 Washburn University, Topeka KS, USA; 3 Mansfield Orthopedics, 555 Washington Hwy, 05661 Morrisville, VT, USA

**Keywords:** Office arthroscopy, Costs of care, MRI, Surgical arthroscopy

## Abstract

**Background:**

The purpose of this analysis was to determine whether in office diagnostic needle arthroscopy (Visionscope Imaging System [VSI]) can provide for improved diagnostic assessment and; more cost effective care.

**Methods:**

Data on arthroscopy procedures in the US for deep seated pathology in the knee and shoulder were used (Calendar Year 2012). These procedures represent approximately 25-30% of all arthroscopic procedures performed annually. Sensitivities, specificities, positive predictive, and negative predictive values for MRI analysis of this deep seated pathology from systematic reviews and meta-analyses were used in assessing for false positive and false negative MRI findings. The costs of performing diagnostic and surgical arthroscopy procedures (using 2013 Medicare reimbursement amounts); costs associated with false negative findings; and the costs for treating associated complications arising from diagnostic and therapeutic arthroscopy procedures were then assessed.

**Results:**

In patients presenting with medial meniscal pathology (ICD9CM diagnosis 836.0 over 540,000 procedures in CY 2012); use of the VSI system in place of MRI assessment (standard of care) resulted in a net cost savings to the system of $151 million. In patients presenting with rotator cuff pathology (ICD9CM 840.4 over 165,000 procedures in CY2012); use of VSI in place of MRI similarly saved $59 million. These savings were realized along with more appropriate care as; fewer patients were exposed to higher risk surgical arthroscopic procedures.

**Conclusions:**

The use of an in-office arthroscopy system can: possibly save the US healthcare system money; shorten the diagnostic odyssey for patients; potentially better prepare clinicians for arthroscopic surgery (when needed) and; eliminate unnecessary outpatient arthroscopy procedures, which commonly result in surgical intervention.

## Background

Magnetic resonance imaging (MRI) or arthrography (MRA) and clinical evaluation are the tools most commonly used to assess soft tissue injuries to the shoulder and knee joints. However, MRI (A) assessment has associated drawbacks, including a relatively high incidence of false negative [FN] (i.e. Pathology shown to be negative on MRI when in actuality pathology is present) and false positive [FP] (i.e. Pathology shown to be present on MRI when in actuality there is none) findings. The high incidence of FP and FN occurs most commonly with deep intra-articular structures such as the medial meniscus of the knee and with the rotator cuff. In the US, for the year 2012, over 540,000 procedures for tears of the medial meniscus of the knee and; over 165,000 procedures for tears of the rotator cuff were performed. In systematic reviews and meta-analyses of MRI diagnostic findings for these types of lesions the sensitivity, specificity, positive predictive value, and negative predictive values were found to be 91.4%, 81.1%, 83.2%, and 90.1% for medial meniscal tears [1]. Further, in similar systematic reviews for internal lesions of the shoulder [2] sensitivities and specificities were found to be 85.5% and 90.4% respectively for partial or full thickness rotator cuff tears. The data used in these meta-analyses were mainly from academic institutions where MRI assessment/accuracy of diagnosis is typically better than what is seen in the community setting (where most MRIs are performed) [3-6]. What these sensitivities and specificities mean in knee pathology is that in approximately one out of every 5 cases (or 100% less 81% specificity value above = 19%; which is the FP value) of a positive MRI finding for a medial meniscus lesion, there may not be a lesion present. Therefore, in approximately 20% of the 540,000 arthroscopies performed (or 108,000 arthroscopies), an unnecessary procedure is likely performed. It has been noted that medial meniscal tears are the hardest pathology to diagnose with accuracy [7]. Additionally in approximately 1 out of every 10 cases of medial meniscal tears (100% less 91.4% sensitivity value above = 8.6%; which is the FN value) a lesion was actually present when there was a negative finding on MRI. Therefore 10% of patients are left to deal with the pain and disability associated with untreated pathology. These types of MRI findings often leave clinicians wondering whether they should proceed with arthroscopy due to: uncertainty in the MRI finding; concern over medico-legal reasons and; a desire to help the patient. In practice, many clinicians perform the arthroscopy. Additionally, there are suggestions in the literature that MRIs are minimally influential in altering the treatment plan of the clinician [8-11]. Further, clinicians may view MRI findings with skepticism based on the MRI findings underestimating deep intra articular defects [12-14]. These recommendations and clinician skepticism are reflected in the following fact: 99% of arthroscopies in the US are therapeutic in nature [15]. The question is whether these therapeutic arthroscopies are really necessary. Unnecessary care/procedures are by definition substandard or poor quality of care [16]. An alternative more accurate diagnostic modality might mitigate some of the above issues.

Small bore (needle arthroscopy) has been available for a number of years and appears to be well established in its efficacy. The results reported on the literature have demonstrated similar accuracy and complications to standard, larger sized arthroscopes – for knee, shoulder, and other joints [17-27].

A newer and smaller bore arthroscope with improved optics and visualization, the VisionScope System (VSI), was recently introduced which enables office-based pre-operative and post-operative arthroscopic imaging and diagnostics. These types of procedures include: diagnostics of joints such as the knee, shoulder, ankle, wrist, hip and elbow. The key component of this system is a proprietary 1.4 mm diameter semi-rigid/fiber-lens endoscope system, which is small enough to fit through an arthrocentesis needle. The VSI technique is described in Appendix 1.

The VSI provides a minimally invasive option with only one entry portal. It does not require general anesthesia, irrigation fluid, and is disposable. The advantages of the VSI are that patients gain a diagnosis during their first office visit and can eliminate the need for a diagnostic MRI. Overall, it can create a “better” experience for the patient, with fewer office visits, a timelier and definitive diagnosis and treatment plan, and lower health care costs.

To date no one had examined the economic effect of more widespread use of office based needle arthroscopy products. The purpose of this analysis is to examine the overall costs (including a cost assessment of morbidity and mortality based on use of needle arthroscopy and follow on surgical arthroscopy) to the US health care system of a needle arthroscope such as the VSI compared to current diagnostic and treatment modalities offered (defined as standard of care or SOC) [28]. The hypothesis is that with use of the VSI office arthroscopy system, significant savings to the system might be realized when compared to the current standard of care.

## Methods

Data sources:

• National Survey Ambulatory Surgery 2006 [29]. This survey, conducted periodically by the National Center for Health Statistics (NCHS), covers ambulatory surgery procedures performed in hospitals and freestanding ambulatory surgery centers in the United States. The hospital universe includes non institutional hospitals exclusive of Federal, military, and Department of Veterans Affairs hospitals, located in the 50 States and the District of Columbia. These numbers of procedures for medial meniscal tears and rotator cuff tears were inflated to 2012 using annual growth rates of 6.9% and 7.6% respectively based on historical growth rates in these types of procedures [15].

• Sensitivity and specificity estimates for shoulder and knee arthroscopy were derived from systematic reviews and meta-analyses as described above [1,2]. The analysis of FP and FN findings using MRI as part of SOC can be found in Additional file 1.

• Costs for diagnostic and treatment paradigms using current methods and for the VSI are Medicare’s national average actual reimbursement rates for each procedure (unless otherwise specified). Costs for complications or unnecessary care associated with either method were estimated using either current national average Current Procedural Terminology (CPT) [physician work component], Ambulatory Payment Classification (APC) [hospital outpatient procedure] or Diagnostic Related Group (DRG) [hospital inpatient procedure] associated with treating complications or unnecessary care. Costs were calculated for both MRI and VSI positive and negative findings.

• Estimates for complications (morbidity and mortality) from each diagnostic method were derived from the literature and are outlined below along with their sources.

• Direct experience in using the VSI system.

Direct cost calculation:

• The following diagnostic and treatment paradigms were used for both current (MRI) and VSI [all assumed to be performed in either the physician office or outpatient settings]: 836.0 (tear of medial cartilage or meniscus of knee); 840.4 (partial or full thickness tears of the rotator cuff). The reasons for using these particular diagnoses were that they represent a large portion of all US therapeutic arthroscopic knee (32%) [30] and shoulder (26%) [31] procedures and; represent pathology where MRI may not provide accurate diagnoses (i.e. high FP and FN rates). The costs for these diagnoses and treatments (including complications [morbidity and mortality] arising from the procedure) were assessed over the acute time period (through the follow up visit post procedure) in the following manner:

• For the knee:

SOC: Orthopedic consult (using CPT 99203 – Evaluation and Management [E & M] for a new patient) + Xray (CPT 73560 – radiologic exam 1 or 2 views) + MRI (CPT 73721 – MRI any lower extremity joint - Global) + MRI (CPT 73721–26 – MRI any lower extremity joint – Professional) + Arthrocentesis (CPT 20610 – aspiration or injection of a major joint @ 10% of time [32]) + Hospital Outpatient Arthroscopy [assumes a chondroplasty was performed when a patient was diagnosed accurately for pathology [i.e. a TP] – CPT 29877 and; the most conservative treatment was employed – i.e. meniscectomy only - CPT 29881 when diagnosed inaccurately for pathology [i.e. a FP]] + CPT 01440 (anesthesia @ 45 minutes) + Follow up orthopedic consult (if MRI is positive or negative) (CPT 99213 – E & M existing patient).

VSI: Orthopedic consult (using CPT 99203 – Evaluation and Management for a new patient) + Xray (CPT 73560 – radiologic exam 1 or 2 views) + VSI (CPT 29870 nonfacility) + Hospital Outpatient Arthroscopy [assumes a chondroplasty was performed when a patient was diagnosed accurately for pathology [i.e. a TP] – CPT 29877] + CPT 01440 (anesthesia @ 45 minutes) + Follow up orthopedic consult (if VSI is positive) (CPT 99213 – E & M existing patient).

• For the shoulder:

SOC: Orthopedic consult (using CPT 99203 – Evaluation and Management [E & M]) + MRI (CPT 73221 – MRI any upper extremity joint - Global) + MRI (CPT 73221–26 – MRI any upper extremity joint – Professional) + Arthrocentesis (CPT 20610 – aspiration or injection of a major joint @ 8% of time [33]) + Hospital Outpatient Arthroscopy [CPT 29827 – shoulder, rotator cuff repair] + CPT 01630 (anesthesia @ 90 minutes) + Follow up orthopedic consult (if MRI is positive or negative) (CPT 99213 – E & M).

VSI: Orthopedic consult (using CPT 99203 – E & M) + VSI (CPT 29805) + Hospital Outpatient Arthroscopy [CPT 29827 – arthroscopy with rotator existing patient) cuff repair] + CPT 01630 (anesthesia @ 90 minutes) + Follow up orthopedic consult (if VSI is positive) (CPT 99213 – E & M).

• The costs for each of the above services/procedures are (2013 Medicare reimbursement data) (Table 1). This consists of:

1) Overlay of the incidence and costs for complications associated with each procedure above and treated in the appropriate care setting. The complications evaluated were those seen with standard arthroscopy. The complication rates used for knee arthroscopy were derived from a recent article (2011) appearing in the Journal of Bone and Joint Surgery [34] (Table 2). The complication rates applied to the costs of shoulder arthroscopy were also derived from the literature [35,36] (Table 3). Since the VSI disposal arthroscope is similar in size to needles used in arthrocentesis, the complication rates applied to the costs of diagnostic knee & shoulder arthroscopy using VSI were derived from the literature based on the types of complications seen with arthrocentesis (Table 4).

2) False positive results on MRI examination were assumed to be treated via a surgical arthroscopic procedure. False positive results were assumed not to be treated upon VSI examination.

3) False negative results on MRI were assumed to be treated first via physical therapy [PT] (CPT 97110; twice per week for 6 weeks [37] and; at an adherence rate/attendance of 86% [38] of these visits or; 10.3 visits [12 × 0.86]) and then via an arthroscopic procedure based on crossover rates from randomized controlled trials [37,39]. The PT sessions as per above are consistent with how private payers such as CIGNA cover and pay for physical therapy [40]. This cross over from PT to a therapeutic arthroscopy procedure varied from 22% for the shoulder [39] to 30% for the knee [37]. If PT crossed over to a therapeutic procedure, the procedural codes used for the knee were: CPT 29881, APC 0041, and CPT 99213 and; for the shoulder: CPT 29827, APC 0042 and CPT 99213. Physical therapy was assumed to occur for a very large portion of these patients (85%); with an assumption that 15% did not have insurance [41] and; that lack of insurance was a predisposing factor to not undergoing PT [42]. The number of sessions [10.3 over a 6 week period] was reflective of above [38].

4) True positives and FPs on MRI were assumed to undergo PT post surgical arthroscopy procedure and; FN crossover (FN CO) patients who underwent surgery were also assumed to undergo PT. True positive and FN patients with VSI were assumed to undergo PT post surgical arthroscopy. The same assumptions were made for VSI regarding number of PT sessions.

**Table 1:** Cost inputs used in diagnostic and therapeutic procedures

**Procedure code**	**Description**	**2013 Medicare reimbursement**
CPT 99203	E & M new patient – 30 minutes (nonfacility [NF])	$108.19
CPT 73560	Xray knee one or two views	$32.32
CPT 73721	MRI knee - Global	$405.21
CPT 73721-25	MRI knee - Professional	$66.69
CPT 73221	MRI shoulder - Global	$405.21
CPT 73221-26	MRI shoulder – Professional	$66.69
CPT 29805	Diagnostic shoulder arthroscopy (NF)	$479.38
CPT 29827	Rotator cuff repair	$1,086.35
CPT 29870	Diagnostic knee arthroscopy (NF)	$603.23
CPT 29877	Chondroplasty (Facility) - if a TP or a FN crossover (FN CO)	$632.49
CPT 29881	Meniscectomy (Facility) – if a FP	$551.51
CPT 01440	General anesthesia (45 minutes) – for hospital outpatient procedure - knee	$131.55
CPT 01630	General anesthesia (90 minutes) for hospital outpatient procedure – rotator cuff repair	$243.32
APC 0041	Outpatient knee arthroscopy	$2,111.62
APC 0042	Outpatient shoulder arthroscopy	$3,880.22
CPT 99213	E & M existing patient – 30 minutes Non-facility (NF)	$72.81
CPT 97110	Therapeutic procedures, 15 minutes each, physical therapy	$31.98
CPT 20610	Arthrocentesis – major joint	$65.56

**Table 2:** Complications, incidence, and costs applied diagnostic knee arthroscopy

**Complication**	**Incidence **[25]	**Cost for treatment**
Re-operation (any reason) (including infection)	0.30% (3 out of 1,000)	- CPT 29871 (surgical, for infection, lavage and drainage); $521.91
		- APC 0041(knee arthroscopy/drainage); $2,111.62
Venous thromboembolism (VTE)	0.19% (1.9 out of 1,000)	Costs over a 12 month timeframe for treating VTE; $14,865
Deep vein thrombosis (DVT)	0.12% (1.2 out of 1,000)	Costs over a 12 month timeframe for treating DVT [43]; $14,865
Pulmonary embolism (PE)	0.08% (0.8 out of 1,000)	Costs over a 12 month timeframe for treating PE [43]; $22,900

**Table 3:** Complications, incidence, and costs applied to shoulder arthroscopies

**Complication**	**Incidence **[25]	**Cost for treatment**
Venous thromboembolism (VTE)	0.038% (0.4 out of 1,000)	Costs over a 12 month timeframe for treating VTE; $14,865
Deep vein thrombosis (DVT)	0.029% (0.3 out of 1,000)	Costs over a 12 month timeframe for treating DVT; $14,865
Pulmonary embolism (PE)	0.017% (0.2 out of 1,000)	Costs over a 12 month timeframe for treating PE; $22,900
Arthrofibrosis	1% (10 out of 1,000)	CPT 29825 (lysis of adhesions): $594.38; APC 0042: $3880.22
Repeat surgical intervention procedure	0.6% (6 out of 1,000)	CPT 29827 (rotator cuff repair): $1,086.35; APC 0042: $3,880.22
Deep Infection	0.2% (2 out of 1,000)	DRG 863 – medical treatment post op infection: $5,665
Bicep tendon rupture	0.2% (2 out of 1,000)	DRG 507: Major Shoulder/Elbow
		Procedures or Other Upper
		Extremity Procedures with CC: $10,790 + CPT 23410: $831

**Table 4:** Complications from arthrocentesis

**Complication**	**Incidence **[25]	**Cost for treatment**
Re-operation (any reason) (including infection)	0.01% (1 out of 10,000)	− CPT 29871 (surgical, for infection, lavage and drainage); $521.91
		− APC 0041(knee arthroscopy/drainage); $2,111.62

## Results

### Knee arthroscopy analysis of costs SOC versus VSI

Based on the sensitivity, specificity, positive and negative predictive values obtained from the literature [1] it was found that in order for 540,803 procedures to be performed (per methodology above using NSAS data, there were 362,000 medial meniscus procedures performed in 2006 for IC9CM diagnosis 836.0. This figure was compounded annually at 6.9% for 6 years which resulted in 540,803 procedures for CY 2012) (both true positives [450,172] and false positive [90,631] MRI findings resulting in procedures = 540,803). Further, 431,523 negative findings for MRI also resulted (both false negatives and true negatives). The incidence of positive and negative MRI diagnosis and resultant treatment of this condition therefore totaled 972,326 (540,803 + 431,523) patients.

For medial meniscal tears, the overall cost savings per patient using VSI was $155 ($3,026 less $2,871) and the overall savings to the health care system equated to $151 million ($2,943 million less $2,792 million) (Table 5). (For more details on the costs see Additional file 2).

**Table 5:** Diagnosis & treatment of medial meniscal tears (MMT) and rotator cuff tears (RCT) using SOC and VSI

**Modality**	**# patients**	**Tot cost/pt for diagnostic and treatment paradigm**	**Cost of complications per patient due to exposure to arthro procedure**	**Total costs to system (millions)**
SOC (MMT)	972,326	$3,026	$72	$2,943
VSI (MMT)	972,326	$2,871	$43	$2,792
Savings using VSI (MMT)		$155	$29	$151
SOC (RCT)	429,502	$3,290	$146	$1,438
VSI (RCT)	429,502	$ 3,118	$92	$1,379
Savings using VSI (RCT)		$162	$54	$59

### Shoulder arthroscopy analysis of costs SOC versus VSI

Based on the sensitivity, specificity, positive and negative predictive values obtained from the literature [2] it was found that in order for 166,191 procedures to be performed (both true positives and false positive MRI findings), 263,311 negative findings for MRI also resulted (both false negatives and true negatives). (Per the methodology above using NSAS data, there were 108,000 rotator cuff procedures performed in 2006 for IC9CM diagnosis 840.4. This figure was compounded annually at 7.6% for 6 years which resulted in 166,191 procedures for CY 2012.) The incidence of positive and negative MRI diagnosis and resultant treatment of this condition therefore totaled 429,502 (166,191 + 263,311) patients.

For rotator cuff tears, the overall cost savings per patient using VSI was $138 ($3,348 less $3,210) and the overall savings to the health care system was $59 million ($1,438 million less $1,379 million) (Table 5). [See Additional file 3 for more detail].

Total savings to the healthcare system by procedure are shown in Table 5. This amount adds up to $210 million ($151 million + $59 million) for these 2 procedures alone.

## Discussion

There is a high FP rate of intra-articular MRI findings in deep knee and shoulder pathology – such as in medial meniscus and rotator cuff tears. This can result in clinicians performing procedures that may be unnecessary in a significant number of knee and shoulder pathology due to an inability of the MRI to accurately assess this intra-articular pathology. Additionally, there is a “not insignificant” number (approximately 1 in 10) of MRIs that result in FN findings – which also can result in care that may not be needed as a first course of action (e.g. physical therapy and cross over to therapeutic procedures). This analysis examined the issues of FP and FN MRI findings on the potential downstream care associated with MRI findings and found that the excess costs in unnecessary care (from MRI assessment) approached $210 million. False positive MRI findings expose patients to potentially unnecessary arthroscopic surgeries and their concomitant risks and; since patients are also misdiagnosed with MRI as FN (not having a lesion when in fact they do) – undergo more care than may be necessary.

Another potential positive outcome of using a VSI like technology, not discussed above, is a potential shortening of the patient’s diagnostic odyssey. With such a system the patient is awake and can view and review their pathology actively with the physician. This also engages the patient in the course of diagnosis and potential treatment and; may reduce any patient anxiety that results from not knowing.

The use of in-office arthroscopy may also assist the physician in their preparation (based on better and more accurate information) for surgical intervention (when needed). There is likely a benefit of being better prepared as; the clinician can plan and prepare for the appropriate surgical intervention. An example of this is allograft tissue which must be procured ahead of a surgical procedure; the use of which cannot be determined inter-operatively. While not quantified above, this may also shorten the procedure time, outcome, and costs of the therapeutic procedure.

The issue of unnecessary and inefficient use of services in healthcare is a major concern for policy makers and payers [44]. Use of the VSI system in the physician office setting may help mitigate unnecessary care, as it can help reduce the number of FP and FN findings seen on MRI and thus; reduce the need for clinicians to perform “definitive diagnostic” arthroscopy procedures with FP and; any follow on care resulting from FN findings. Most troubling however, is the finding that 99% of all arthroscopic procedures end up being surgical in nature. Based on meta-analysis findings, 15-20% of procedures should likely be diagnostic only (i.e. those FP findings).

### Limitations

The following limitations of the above analysis should be taken into account:

• It assumes the most conservative treatment when a FP result was treated under SOC with knee procedures (i.e. meniscectomy versus repair). This may have underestimated the complication rates and costs reported on above with SOC as; more aggressive treatment may have been undertaken with these FP findings.

• It assumes that false positive MRI results always end up as a surgical arthroscopy procedure. This may not always be the case. However, in pathology that appears positive under MRI and is difficult to interpret as in the pathology described herein (e.g. deep tissue, repeat procedures), surgical arthroscopy is commonly performed. This in turn may have overestimated the actual costs of the MRI paradigm. Since MRI is used in conjunction with a physical exam, especially in complex injuries like those described above, surgical arthroscopy may be lower than what is evaluated on in this paper. However, it also should be noted that MRI diagnosis of meniscal tears, appears to be more accurate (using fitted Receiver Operating Characteristic [ROC] curves) versus physical examination. Thus the combination of the two (MRI plus physical examination) may not necessarily be more accurate than MRI alone [44].

• The results are likely conservative for cost savings with VSI than what would be found in the community setting. In community settings the sensitivities and specificities of MRI findings are less accurate [3-6] than what is found in academic medical centers (where the sensitivities and specificities reported on in this paper were derived). This improvement in accuracy is due to better trained clinicians in academic medical centers and better MRI equipment. Therefore, more patients would likely be treated with unnecessary procedures (based on FP findings) and the concomitant costs would be higher.

• It assumes that false positive MRI results, upon a VSI finding were not treated. This is not always the case. Although unlikely, VSI may also result in a false positive finding – resulting in a surgical arthroscopy procedure. This may have underestimated the actual costs under the VSI diagnostic/treatment paradigm.

• It focuses on difficult to diagnose conditions with MRI (i.e. intra-articular pathology) where a product such as VSI would likely have the most clinical utility. This is only a part (~30%) of the annual number of surgical arthroscopic procedures performed in the US. If the VSI diagnostic procedure were performed prior to other deep seated pathology in the knee and shoulder (e.g. lateral meniscus tears and glenoid/labral lesions) additional and significant savings would be realized. It was the intention of this analysis to determine if any savings could be realized in common procedures.

• Since no “good” data exist in the literature on the complication rates seen with small needle/single access site arthroscopy, the complication rate assumed in this analysis with the VSI procedure was an estimate based on ½ of what the complication rate would be with arthroscopy (i.e. single access versus two access sites with traditional arthroscopy). In reports of case series on small needle arthroscopy, none of the complication rates noted above were seen [45]. The only estimated complication used with VSI was for infections at a rate of 0.01%. This was based off of the infection rate found when performing office-based arthrocentesis – which has a needle size similar to that of the VSI. We believe this complication rate is conservative in nature.

• The use of a full course of PT (6 weeks and 10 visits @ $1,320) was assumed to be 85% in the FN, FN CO, TP, and FP SOC patients and; at 85% in the TP and FN VSI patients. The assumption was that since an orthopedist prescribed the PT, it was closely adhered to [38,42]. However, 85% adherence to PT may have not been the case and as well, some patients may have taken it upon themselves to rehab at home and thus may not have incurred this cost. Therefore the PT costs may have been overestimated.

• This evaluation and the safety and efficacy of VSI does not include patients who may present with acute hemarthrosis.

• It assumes Medicare savings only. Since private pay in the US (e.g. BCBS) commonly pays 15-20% higher than Medicare and most patients who are evaluated for meniscal and rotator cuff damage are <65 years of age, the cost savings to the US health care system may be higher.

• The analysis assumes 100% sensitivity and specificity of small bore arthroscopy to traditional arthroscopy. This may not be the case. Additionally, traditional arthroscopy is not perfect in its assessment of pathology.

• For patients who are highly likely to have negative pathology, the preference might be for a non-invasive test such as an MRI versus VSI. Thus the overall costs savings using VSI versus MRI might be mitigated due to this.

## Conclusion

Utilization of the VSI system can reduce costs. The cost of a VSI diagnostic and treatment paradigm is less than a similar MRI paradigm on a per patient basis for both shoulder and knee pathology (by $138-$155 respectively) and; many arthroscopies for FPs are eliminated along with their concomitant risks. These cost savings may translate to other countries that perform arthroscopy procedures in similar settings (e.g. hospital outpatient).

## Appendix 1: Vision scope arthroscopic technique

### Knee

The procedure is an in-office diagnostic technique performed in a clinical exam room. The patient is brought into an exam room with their legs hanging over the table and lying supine with their hands on their stomach. The surgeon drapes the knee with sterile drapes and uses Betadine to clean the area. The joint is then injected with 5–10 ccs of Lidocaine to use as a local anesthetic. Once the area is numb, a sharp trocar is used to insert the cannula into the joint to an initial depth of 45 mm parallel to the tibial midline and flush with the surface of the proximal tibia. Once inside the joint, 1.44 mm diameter camera is inserted into the cannula. A diagnostic arthroscopy is performed. During the procedure, 1 cc increments of sterile saline are injected into the joint to clear the view. Patients are able to see real time diagnostic images during their procedure.

### Shoulder

The patient is brought into an exam room with their legs hanging over the table and the patient-sitting upright on the exam table. The surgeon drapes the shoulder with sterile drapes and uses Betadine to clean the area. A 22–25 gauge needle with a 10 cc syringe is used to administer analgesia. Then 1–2 ccs of Lidocaine is injected into the joint and 8–9 ccs is fanned out in the capsule and soft tissue around the insertion point. Once the area is numb, a sharp trocar is used to insert the cannula into the joint about 1.5 cm below the lateral corner of the acromion (inferior to posterolateral corner of acromion) through a transcuff portal. Once inside the joint, 1.44 mm diameter camera is inserted into the cannula. A diagnostic arthroscopy is performed.

## Competing interests

Jeff Voigt works as a reimbursement consultant for Visionscope Technologies, an office based arthroscopy technology mentioned in the manuscript.

Michal Mosier, PhD has not competing interests.

Bryan Huber, MD is user of the Visionscope arthroscopy technology.

## Authors’ contributions

JV, MM, BH developed the hypothesis and laid out the methodology for testing the hypothesis. JV and BH developed the first draft of the manuscript. MM provided the appropriate statistical analysis. JV performed the literature search for relevant articles. MM extensively reviewed the first draft and made edits. MM further reviewed the statistics and ensured the integrity of the data. JV, MM, BH read and approved the final manuscript.

## Pre-publication history

The pre-publication history for this paper can be accessed here:

http://www.biomedcentral.com/1472-6963/14/203/prepub

## Supplementary Material

Additional file 1 Sensitivity, specificity, positive predictive value and negative predictive values. Click here for file

Additional file 2 Cost analysis SOC versus VSI – Medial meniscal diagnosis, therapy and surgery. Click here for file

Additional file 3 Cost analysis SOC versus VSI – rotator cuff diagnosis, therapy, and surgery. Click here for file
