# Supplementary material for: In-office diagnostic arthroscopy for knee and shoulder intra-articular injuries its potential impact on cost savings in the United States
Source: BMC Health Serv Res. 2014 May 5;14:203. doi: 10.1186/1472-6963-14-203 (PMC4101857; doi:10.1186/1472-6963-14-203)
Supplement: Additional file 1 — Sensitivity, specificity, positive predictive value and negative predictive values. [file 1472-6963-14-203-S1.docx]

**Additional file 1: Sensitivity, specificity, positive predictive value and negative predictive values**

| **Arthroscopy procedures for medial meniscal tears - Diagnosis code 836.0.** | | | | | | | | | | | | |  |
| --- | --- | --- | --- | --- | --- | --- | --- | --- | --- | --- | --- | --- | --- |
| **Assumes that all positive findings (TP, FP) on MRI were followed up with an arthroscopy procedure** | | | | | | | | |  | |  | |  |
|  |  |  |  |  |  |  |  | |  | |  | |  |
| **SOC** | **Definite** |  | **Suspected** |  |  |  |  | |  | |  | |  |
|  | **probable** |  | **not present** |  | **Sum** | Positive |  | |  | |  | |  |
|  | (all positives that should be positive) |  | (all negatives that should be negative) |  |  | Pred val |  | |  | |  | |  |
| Medial meniscal tear as primary diagnosis (836.0) and arthroscopy procedures performed 2012 | 450,172 | TP | 90,631 | FP | 540,803 | 83.2% |  | |  | |  | |  |
|  |  |  |  |  |  |  |  | |  | |  | |  |
|  |  |  |  |  |  | Negative |  | |  | |  | |  |
|  |  |  |  |  |  | Pred val |  | |  | |  | |  |
|  | 42,518 | FN | 389,005 | TN | 431,523 | 90.1% | |  | |  | |  | |
|  |  |  |  |  |  |  |  | |  | |  | |  |
| Sum | 492,690 |  | 479,636 |  |  |  |  | |  | |  | |  |
|  |  |  |  |  |  | Accuracy |  | |  | |  | |  |
| Sensitivity = | 91.4% | TP/Sum | 81.1% | TN/Sum | = Specificity | 86.3% |  | |  | |  | |  |

| **Arthroscopy procedures for rotator cuff repair - Diagnosis code 8404- partial or full thickness tears of the rotator cuff** | | | | | | | | |
| --- | --- | --- | --- | --- | --- | --- | --- | --- |
| **Assumes that all positive findings (TP, FP) on MRI were followed up with an arthroscopy procedure** | | | | | | | |  |
|  |  |  |  |  |  |  |  |  |
|  | **Definite** |  | **Suspected** |  |  |  |  |  |
|  | **probable** |  | **not present** |  | **Sum** | Positive |  |  |
|  | (all positives that should be positive) |  | (all negatives that should be negative) |  |  | Pred val |  |  |
| Rotator cuff as primary diagnosis (8404-) and arthroscopy procedures performed 2012 | 140,763 | TP | 25,427 | FP | 166,191 | 84.7% |  |  |
|  |  |  |  |  |  |  |  |  |
|  |  |  |  |  |  | Negative |  |  |
|  |  |  |  |  |  | Pred val |  |  |
|  | 23,872 | FN | 239,439 | TN | 263,311 | 91.0% |  |  |
|  |  |  |  |  |  |  |  |  |
| Sum | 164,636 |  | 264,866 |  |  |  |  |  |
|  |  |  |  |  |  | Accuracy |  |  |
| Sensitivity = | 85.5% | TP/Sum | 90.4% | TN/Sum | = Specificity | 88.5% |  |  |
