# Supplementary material for: In-office diagnostic arthroscopy for knee and shoulder intra-articular injuries its potential impact on cost savings in the United States
Source: BMC Health Serv Res. 2014 May 5;14:203. doi: 10.1186/1472-6963-14-203 (PMC4101857; doi:10.1186/1472-6963-14-203)
Supplement: Additional file 2 — Cost analysis SOC versus VSI – Medial meniscal diagnosis, therapy and surgery. [file 1472-6963-14-203-S2.docx]

Additional file 2: Cost analysis SOC versus VSI – Medial meniscal diagnosis, therapy and surgery

| **Diagnosis and treatment for medial meniscal tear [knee] - ICD9CM Diagnosis code 836.0** | | |  | |  | |  |  |
| --- | --- | --- | --- | --- | --- | --- | --- | --- |
| Standard of Care versus VSI diagnosis and treatment paradigm - costs using 2013 reimbursement data | | | | |  | |  |  |
| Number of diag. & ther. procedures performed for ICD9CM Diagnosis code 836.0 = | | 540,803 | derived from positive findings (TP and FP) | | | | |  |
| Number of diagnostic procedures performed for ICD9CM Diagnosis code 836.0 = | | 431,523 | derived from negative findings (FN and TN) | | | | |  |
| **Procedure code** | **Description** |  | **SOC Cost** | **Notes** | | **VSI Cost** | | **Notes** |
| CPT 99203 | Evaluation and management - new patient - 30 minutes |  | $108.19 |  | | $108.19 | |  |
| CPT 73560 | Xray 1-2 views |  | $32.32 |  | | $32.32 | |  |
| CPT 73721 | Magnetic resonance (e.g., proton) imaging, any joint of lower extremity; without contrast material, non-facility (RVUs of 11.91) (Global) |  | $405.21 |  | | $0.00 | |  |
| CPT 73721-26 | Magnetic resonance (e.g., proton) imaging, any joint of lower extremity; without contrast material, non-facility (RVUs of 1.96) (Professional component "-26") |  | $66.69 |  | | $0.00 | |  |
| CPT 20610 | Arthrocentesis - aspiration or injection major joint or bursa @ 10% of time for diagnosis (1) |  | $65.56 |  | | $0.00 | |  |
| CPT 29870 | Arthroscopy, knee, diagnostic, with or without synovial biopsy (separate procedure) - nonfacility setting |  | $0.00 |  | | $603.23 | |  |
| CPT 29877 | Arthroscopy, knee surgical; chondroplasty – debridement or shaving of articular cartilage [for TP findings] |  | $632.49 |  | | $632.49 | |  |
| CPT 29881 | Arthroscopy, knee, surgical; with meniscectomy (medial OR lateral, including any meniscal shaving) including debridement/shaving of articular cartilage (chondroplasty), same or separate compartment(s), when performed [for FP and FN CO findings] |  | $551.51 |  | | $551.51 | |  |
| CPT 01440 | General anesthesia @ 45 minutes |  | $131.55 |  | | $131.55 | |  |
| APC 0041 | Hospital outpatient - knee arthroscopy/surgery |  | $2,111.62 |  | | $2,111.62 | |  |
| CPT 99213 | Evaluation and management - existing patient - 30 minutes |  | $72.81 |  | | $72.81 | |  |
| Total cost per patient (for true positive findings) | |  | $3,567.44 |  | | $3,692.21 | |  |
| Total cost per patient (for false negative findings) | |  | $3.486.46 |  | | $0 | |  |
| Total cost per patient (for neg findings) [diagnostic procedures only) | |  | $618.97 |  | | $743.74 | |  |
| Number of surgical procedures performed based on TP diagnostic findings = | |  | 450,172 | (TP ) | | 450,172 | | (TP) |
| Number of surgical procedures performed based on FP diagnostic findings = | |  | 90,631 | (FP) | | 0 | | (FP) |
| Number of surgical procedures performed based on FN MRI findings = | |  | 0 |  | | 42,518 | | (FN) |
| Number of people who are medically managed (e.g. PT) due to FN MRI findings= | |  | 42,518 | (FN) | | 0 | |  |
| Number of diag procedures performed based on MRI neg findings = | |  | 431,523 | (FN+TN) | | 479,636 | | (FP+TN) |
| Total cost to system for diag and treatment of all positives that should be pos = | |  | $1,605,959,799 | (TP ) | | $1,819,114,945 | | (TP + FN) |
| Total cost to system for diagnosis and treatment FP = | |  | $315,979,903 | (FP) | | $0 | |  |
| Total cost to system for diagnosis = | |  | $267,098,065 | (FN+TN) | | $356,724,479 | | (FP+TN) |
| Cost per patient for medical management [PT](10.3 sessions over 10 wks) = | |  | $1,318 |  | | $1,318 | |  |
| Number FN patients who have insurance (@85% of FN) = | |  | 42,518 | (FN) | | 0 | | (FN) |
| Total costs to system for medical mgmt (physical therapy) of FN findings on MRI = | |  | $56,020,696 | (FN) | | $0 | |  |
| Percent of patients under medical mgmt crossing over to surgery(2) | |  | 30% |  | | 0% | |  |
| Number of patients who crossed over (CO) to surgery in FN group = | |  | 12,755 | (FN CO) | | 0 | |  |
| Cost of FN medical mgmt patients crossing over to surgery = | |  | $37,608,914 |  | | $0 | |  |
| Number of patients who underwent phys ther post surgical arthroscopy (@85%) = | |  | 470,524 | (TP+FP+FN CO) | | 418,787 | | (FN+TP) |
| Costs for patients under physical therapy post surgery for TP, FP, and FN CO results = | |  | $619,951,623 | (TP+FP+FN CO) | | $551,783,042 | | (FN+TP) |
| Total costs diagnosis and treatment (all positive and negative findings)= | |  | $2,902,619,000 |  | | $2,727,622,465 | |  |
| Cost per patient diagnosis & treatment | |  | $2,985 |  | | $2,805 | |  |

| **Complications from arthroscopy procedures** | | **Incidence** | | **Cost/event** | | **Incidence** | | **Cost/event** | |  |  |
| --- | --- | --- | --- | --- | --- | --- | --- | --- | --- | --- | --- |
| Reoperation (any reason) [includes costs of CPT 29871 & APC 0041] | | 0.30% | | $2,633.53 | | 0.010% | | $2,633.53 | |  |  |
| Venous thromboembolism (VTE) [includes 12 month for treatment] | | 0.19% | | $14,865.00 | | 0.127% | | $14,865.00 | |  |  |
| Deep vein thrombosis (DVT) [includes 12 month for treatment] | | 0.12% | | $14,865.00 | | 0.080% | | $14,865.00 | |  |  |
| Pulmonary embolism (PE) [includes 12 month for treatment] | | 0.08% | | $22,900.00 | | 0.053% | | $22,900.00 | |  |  |
| Number of arthro procedures which complications were applied to = | |  | | 553,558 | |  | | 1,465,016 | |  |  |
| **Occurrence of complications based on incidence and number of procedures** | |  | |  | |  | |  | |  |  |
| Reoperation (any reason) [includes costs of CPT 29871 & APC 0041] | |  | | 1,661 | |  | | 147 | |  |  |
| Venous thromboembolism (VTE) | |  | | 1,052 | |  | | 1,860 | |  |  |
| Deep vein thrombosis (DVT) | |  | | 664 | |  | | 1,175 | |  |  |
| Pulmonary embolism | |  | | 443 | |  | | 783 | |  |  |
| **Overall costs for complications** | |  | |  | |  | |  | |  |  |
| Reoperation (any reason) [includes costs of CPT 29871 & APC 0041] = | |  | | $4,373,435 | |  | | $385,816 | |  |  |
| Venous thromboembolism (VTE) = | |  | | $15,634,418 | |  | | $27,646,230 | |  |  |
| Deep vein thrombosis (DVT) = | |  | | $9,874,369 | |  | | $17,460,777 | |  |  |
| Pulmonary embolism = | |  | | $10,141,184 | |  | | $17,923,584 | |  |  |
| Total costs complications = | |  | | $40,023,407 | |  | | $63,425,408 | |  |  |
| Cost complications per patient for those exposed to arthroscopy = | |  | | $72 | |  | | $43 | |  |  |
| Overall costs – diagnosis, therapy, and complications | |  | |  | |  | |  | |  |  |
| **Total costs - diagnosis, therapeutics, and complications** | |  | | $2,942,642,408 | |  | | $2,792,094,053 | |  |  |
| Cost differential complications (which costs more and by how much) = | |  | | $150,548,355 | |  | |  | |  |  |
| Overall cost per patient = | |  | | $3,026 | |  | | $2,871 | |  |  |
| Cost differential per patient (which costs more and by how much) = | |  | | $155 | |  | |  | |  |  |
| **Footnotes:** |  |  |  | |  | |  | |  | |  |
| (1) National Ambulatory Medical Care Survey data 2010 - 10% figure based on 2010 figures for arthrocentesis for meniscal knee injury (100,000/970,000= 10%) | | | | | | | | | | | |
| (2) Katz JN, et al. Surgery versus physical therapy for a meniscal tear and osteoarthrtitis. *New England Journal Medicine* 2013; DOI: 10.1056/NEJMoa1301408 | | | | | | | | |  | |  |
