# Supplementary material for: In-office diagnostic arthroscopy for knee and shoulder intra-articular injuries its potential impact on cost savings in the United States
Source: BMC Health Serv Res. 2014 May 5;14:203. doi: 10.1186/1472-6963-14-203 (PMC4101857; doi:10.1186/1472-6963-14-203)
Supplement: Additional file 3 — Cost analysis SOC versus VSI – rotator cuff diagnosis, therapy, and surgery. [file 1472-6963-14-203-S3.docx]

Additional file 3: Cost analysis SOC versus VSI – rotator cuff diagnosis, therapy, and surgery

| **Diagnosis and treatment for rotator cuff - ICD9CM Diagnosis code 8404** | | |  | | | |  | | |  | | |  | | |  | | |  |  |  |
| --- | --- | --- | --- | --- | --- | --- | --- | --- | --- | --- | --- | --- | --- | --- | --- | --- | --- | --- | --- | --- | --- |
| Standard of Care versus VSI diagnosis and treatment paradigm - costs using 2013 reimbursement data | | | | | | | | | | |  | | |  | | |  | |  |  |  |
| Number of diag & ther. procedures performed for ICD9CM Diagnosis code 8404 = | | | | | 166,191 | | | (derived from TP and FP) | | | | | |  | | |  | |  |  |  |
| Number of diagnostic procedures performed for ICD9CM Diagnosis code 8404 = | | | | | 263,311 | | | (derived from FN and TN) | | | | | |  | | |  | |  |  |  |
| **Procedure code** | **Description** | | |  | | | **SOC Cost** | | | **Notes** | | | **VSI Cost** | | | **Notes** | | |  |  |  |
| CPT 99203 | Evaluation and management - new patient - 30 minutes | | |  | | | $108.19 | | |  | | | $108.19 | | |  | | |  |  |  |
| CPT 73221 | Magnetic resonance (e.g. proton) imaging, any joint of upper extremity; without contrast material, non-facility (RVUs of 11.91) (Global) | | |  | | | $405.21 | | |  | | | $0.00 | | |  | | |  |  |  |
| CPT 73221-26 | Magnetic resonance (e.g. proton) imaging, any joint of upper extremity; without contrast material, non-facility (RVUs of 1.96) (Professional component "-26") | | |  | | | $66.69 | | |  | | | $0.00 | | |  | | |  |  |  |
| CPT 29805 | Arthroscopy, shoulder, diagnostic, with or without synovial biopsy (separate procedure) - nonfacility setting | | |  | | | $0.00 | | |  | | | $479.38 | | |  | | |  |  |  |
| CPT 20610 | Arthrocentesis - aspiration or injection major joint or bursa @ 12.5% of time for diagnosis (1) | | |  | | | $65.56 | | |  | | | $0.00 | | |  | | |  |  |  |
| CPT 29827 | Arthroscopy, shoulder, surgical; with rotator cuff repair | | |  | | | $1,086.35 | | |  | | | $1,086.35 | | |  | | |  |  |  |
| CPT 01630 | General anesthesia @ 90 minutes | | |  | | | $243.32 | | |  | | | $243.32 | | |  | | |  |  |  |
| APC 0042 | Hospital outpatient - rotator cuff repair/surgery | | |  | | | $3,880.22 | | |  | | | $3,880.22 | | |  | | |  |  |  |
| CPT 99213 | Evaluation and management - existing patient - 30 minutes | | |  | | | $72.81 | | |  | | | $72.81 | | |  | | |  |  |  |
| Total cost per patient (for positive findings) | | | |  | | | $5,868.03 | | |  | | | $5,870.27 | | |  | | |  |  |  |
| Total cost per patient (for neg findings) [diagnostic procedures only) | | | |  | | | $585.33 | | |  | | | $587.57 | | |  | | |  |  |  |
| Number of surgical procedures performed based on positive findings = | | | |  | | | 166,191 | | | (TP+FP) | | | 140,763 | | | (TP) | | |  |  |  |
| Number of surgical procedures performed based on FN MRI findings = | | | |  | | | 0 | | |  | | | 23,872 | | | (FN) | | |  |  |  |
| Number of people who are treated (e.g. medical mgmt) due to FN MRI findings= | | | |  | | | 23,872 | | | (FN) | | | 0 | | |  | | |  |  |  |
| Total cost to system for diagnosis and treatment = | | | |  | | | $975,214,571 | | |  | | | $966,451,901 | | |  | | |  |  |  |
| Number of diagnostic procedures performed based on negative findings = | | | |  | | | 263,311 | | | (FN+TN) | | | 264,866 | | | (FP+TN) | | |  |  |  |
| Total cost to system for diagnosis only = | | | |  | | | $154,125,092 | | |  | | | $155,627,316 | | |  | | |  |  |  |
| Cost per patient for medical management [PT](10.6 sessions over 10 wks) = | | | |  | | | $1,318 | | |  | | | $1,318 | | |  | | |  |  |  |
| Number FN patients who had insurance (@85% of FN) = | | | |  | | | 23,872 | | | (FN) | | | 0 | | | (FN) | | |  |  |  |
| Total costs to system for medical mgmt of FN findings on MRI = | | | |  | | | $31,453,174 | | | (FN) | | | $0 | | | (FN) | | |  |  |  |
| Percent of patients under medical mgmt crossing over to surgery(1) | | | |  | | | 22% | | |  | | | 0% | | |  | | |  |  |  |
| Number of patients who crossed over (CO) to surgery in FN group = | | | |  | | | 5,252 | | | (FN CO) | | | 0 | | |  | | |  |  |  |
| Cost of FN medical mgmt patients crossing over to surgery = | | | |  | | | $26,466,017 | | |  | | | $0 | | |  | | |  |  |  |
| Number of patients who underwent phys ther post surgical arthroscopy (@85%) = | | | |  | | | 171,443 | | | (TP+FP+FN CO) | | | 164,636 | | | (FN+TP) | | |  |  |  |
| Costs for patients under physical therapy post surgery for TP, FP, and FN results = | | | |  | | | $225,888,971 | | | (TP+FP+FN CO) | | | $216,920,442 | | | (FN+TP) | | |  |  |  |
| Total costs positive and negative findings = | | | |  | | | $1,413,147,826 | | |  | | | $1,338,999,659 | | |  | | |  |  |  |
| Cost per patient diagnosis & treatment = | | | |  | | | $3,290 | | |  | | | $3,118 | | |  | | |  |  |  |
| **Sources:** | |  | | | |  | | |  | | |  | | |  | | |  | |  |  |
| (1) National Ambulatory Medical Care Survey data 2010 - based on 2010 figures for arthrocentesis of shoulder for rotator cuff lesion (35,000/970,000 = 8%) | | | | | | | | | | | | | | | | | | | | | |
| (2) Brox JI, et al. Arthroscopic surgery versus supervised exercises in patients with rotator cuff disease - prospective RCT with 2.5 year follow-up | | | | | | | | | | | | | | | | | |  | |  |  |
| *Journal Shoulder and Elbow Surgery;1999;*8(2):102-11. | | | | | |  | | |  | | |  | | |  | | |  | |  |  |

| **Complications for arthroscopy procedures** | **Incidence** | **Cost/event** | **Incidence** | **Cost/event** |
| --- | --- | --- | --- | --- |
| Arthrofibrosis [includes CPT 29825 and APC 0042] | 1.00% | $4,474.60 | 0.64% | $4,474.60 |
| Reoperation (any reason) [includes costs of CPT 29827 & APC 0042] | 0.04% | $4,966.57 | 0.01% | $4,966.57 |
| Venous thromboembolism (VTE) [includes 12 month for treatment] | 0.38% | $14,865.00 | 0.24% | $14,865.00 |
| Deep vein thrombosis (DVT) [includes 12 month for treatment] | 0.03% | $14,865.00 | 0.02% | $14,865.00 |
| Pulmonary embolism (PE) [includes 12 month for treatment] | 0.02% | $22,900.00 | 0.01% | $22,900.00 |
| Deep infection [includes DRG 863 - med treatment of post of infection] | 0.20% | $5,665.00 | 0.13% | $5,665.00 |
| Bicep tendon rupture | 0.20% | $11,621.00 | 0.13% | $11,621.00 |
| Number of arthroscopic procedures which complications were applied to = |  | 171,443 |  | 429,501 |
| **Occurrence of complications based on incidence and number of procedures** |  |  |  |  |
| Arthrofibrosis [includes CPT 29825 and APC 0042] |  | 1,714 |  | 2,743 |
| Reoperation (any reason) [includes costs of CPT 29827 & APC 0042] |  | 65 |  | 43 |
| Venous thromboembolism (VTE) [includes 12 month for treatment] |  | 651 |  | 1,042 |
| Deep vein thrombosis (DVT) [includes 12 month for treatment] |  | 50 |  | 80 |
| Pulmonary embolism (PE) [includes 12 month for treatment] |  | 29 |  | 47 |
| Deep infection [includes DRG 863 - med treatment of post of infection] |  | 343 |  | 549 |
| Bicep tendon rupture |  | 343 |  | 549 |
| **Overall costs for complications** |  |  |  |  |
| Arthrofibrosis [includes CPT 29825 and APC 0042] |  | $7,671,381 |  | $12,271,936 |
| Reoperation (any reason) [includes costs of CPT 29827 & APC 0042] |  | $323,563 |  | $213,315 |
| Venous thromboembolism (VTE) [includes 12 month for treatment] |  | $9,684,292 |  | $15,491,997 |
| Deep vein thrombosis (DVT) [includes 12 month for treatment] |  | $739,064 |  | $1,182,284 |
| Pulmonary embolism (PE) [includes 12 month for treatment] |  | $667,427 |  | $1,067,685 |
| Deep infection [includes DRG 863 - med treatment of post of infection] |  | $1,942,447 |  | $3,107,340 |
| Bicep tendon rupture |  | $3,984,674 |  | $6,374,298 |
| Total costs complications |  | $25,012,50 |  | $39,708,855 |
| Cost per patient complication |  | $145.90 |  | $92.44 |
| **Total costs - diagnosis, therapeutics, and complications** |  | $1,438,160,676 |  | $1,378,708,515 |
| Cost differential complications (which costs more and by how much) = |  | $59,452,161 |  |  |
| Overall cost per patient |  | $3,348 |  | $3,210 |
| Cost differential per patient (which costs more and by how much) = |  | $138 |  |  |
